# Supplementary material for: Study on the Cellular Anti-Inflammatory Effect of Torularhodin Produced by Sporidiobolus pararoseus ZQHL Isolated from Vinegar Fungus
Source: Molecules. 2023 Feb 2;28(3):1436. doi: 10.3390/molecules28031436 (PMC9920945; doi:10.3390/molecules28031436)
Supplement: Supplementary file 1 [file molecules-28-01436-s001.zip › molecules-2177758-supplementary.pdf]

Table S1. Conditions in strain enrichment culture

**Time/h**	**1-10**	**10-20**	**20-30**	**30-64**
Temperature /℃	28	28	28	28
pH	6	6	6	6
Ventilation L/min	25	40-50	50	40
Rotating speed /rpm	200-400	600-800	600-800	500-700
Tank pressure /Mpa	0.06	0.08-0.09	0.09-0.1	0.09

Table S2. Sequences of PCR primers.

	Upstream primer （5'-3'）	Downstream primer （5'-3'）
β-actin	CCACAGCTGAGAGGGAAA	AAGGAAGGCTGGAAAAGAGC
TLR4	CTGGGTGAGAAAGCTGGTAA	AGCCTTCCTGGATGATGTTGG
MyD88	GTTGTGTGTGTCCGACCGT	GTCAGAAACAACCACCACCATGC
TNF-α	AGCCCCCAGTCTGTATCCTT	CATTCGAGGCTCCAGTGAAT
IL-1β	GGGCCTCAAAGGAAAGAATC	TACCAGTTGGGGAACTCTGC
COX-2	CCTGAGCATCTACGGTTTGC	ACTGCTCATCACCCCATTCA
iNOS	ACCCAAGGTCTACGTTCAGG	CGCACATCTCCGCAAATGTA
